# Prevalence and Specific Manifestations of Non-alcoholic Fatty Liver Disease (NAFLD) and Diabetes Mellitus Type 2 Association in a Moroccan Population: A Cross-sectional Study

**DOI:** 10.1900/RDS.2022.18.140

**Published:** 2022-09-30

**Authors:** Imane Assarrar, Najoua Messaoudi, Farel Elilie Mawa Ongoth, Wahiba Abdellaoui, Abdessamad Malki, Siham Rouf, Naima Abda, Zahi Ismaili, Hanane Latrech

**Affiliations:** 1 Department of Endocrinology-Diabetology and Nutrition, Mohammed Vi University Hospital Center, Faculty of Medicine and Pharmacy, University of Mohammed First, Oujda, Morocco,; 2 Laboratory of Epidemiology, Clinical Research and Public Health, Faculty of Medicine and Pharmacy, University of Mohammed First, Oujda, Morocco,; 3 Department of Hepatology and Gastroentorology, Mohammed Vi University Hospital Center, Faculty of Medicine and Pharmacy, University of Mohammed First, Oujda, Morocco.

**Keywords:** type 2 diabetes, NAFLD, NASH, insulin resistance, metabolic syndrome

## Abstract

**OBJECTIVE:**

Non-alcoholic fatty liver disease (NAFLD) is recognized as a common cause of chronic liver disease worldwide. Its association with type 2 diabetes mellitus (T2DM) is known to increase the risk of degenerative complications of diabetes and the likelihood of developing severe hepatic injuries. The objective of this study was to assess the prevalence of NAFLD and to describe the characteristics of its association with T2DM.

**METHODS:**

This was a descriptive analytical study, involving patients with T2DM with no history of alcohol consumption, viral hepatitis, hepatotoxic medications, or other chronic liver diseases. The patients underwent an investigation of NAFLD including abdominal ultrasound, non-invasive biomarkers of liver fibrosis, elastography and ultrasound-guided liver biopsy.

**RESULTS:**

We collected data from 180 patients with a mean age of 59.3 ± 10.9 years with strong female predominance. The mean duration of diabetes progression was 9.2 ± 7.3 years. Hepatic sonography showed signs of NAFLD in 45.6% of cases. Non-invasive hepatic biomarkers indicated significant fibrosis in 18.3% of cases. Overall, 21% of patients underwent an elastography evaluation, revealing severe fibrosis or cirrhosis in 15.4% of patients. The diagnosis of NASH (Non-alcoholic steatohepatitis) was confirmed histologically in 3 patients. The overall prevalence of NAFLD was 45.6%. Patients with NAFLD had a statistically significant incidence of obesity, metabolic syndrome, hypertension, dyslipidemia, macrovascular complications, and hypertriglyceridemia (p < 0.05).

**CONCLUSIONS:**

The combination of NAFLD and T2DM is often found in patients with obesity or metabolic syndrome. The presence of NAFLD can be responsible for increased morbidity and important cardiovascular risks in patients with T2DM.

## 1 Introduction

Type 2 diabetes mellitus (T2DM) is a major public health problem. Several studies suggest that type 2 diabetes is associated with non-alcoholic fatty liver disease (NAFLD) [1-6]. NAFLD is recognized as a common cause of chronic liver disease worldwide appearing as a growing public health problem [7]. It is defined by hepatic accumulation of lipids, particularly triglycerides, in the absence of excess alcohol intake with no secondary cause for steatosis [8]. It has a large pathological spectrum, including nonalcoholic fatty liver (NAFL) and nonalcoholic steatohepatitis (NASH).

This sustained liver injury can lead to progressive fibrosis and development of cirrhosis [5,9].

The association of T2DM and NAFLD is known to increase the risk of cardiometabolic complications as well as the likelihood of developing severe hepatic injuries (cirrhosis and hepatocellular carcinoma), and thus, responsible for elevated morbidity and mortality in patients with T2DM [3,10,11]. The prevalence of this association and its characteristics is unknown in Morocco. Therefore, the objective of this study was to assess the prevalence of NAFLD and to describe the specific manifestations of its association with diabetes mellitus type 2 in a developing country’s population.

## 2 Methods

### 2.1 Study design

This was a descriptive and analytical study, with cross-sectional collected data, conducted for a duration of 3 years and 2 months from December 2016 to February 2020, involving patients with type 2 diabetes, followed-up at Mohammed VI University Hospital Center of Oujda, in the eastern portion of Morocco.

### 2.2 Study population

**Figure 1.**
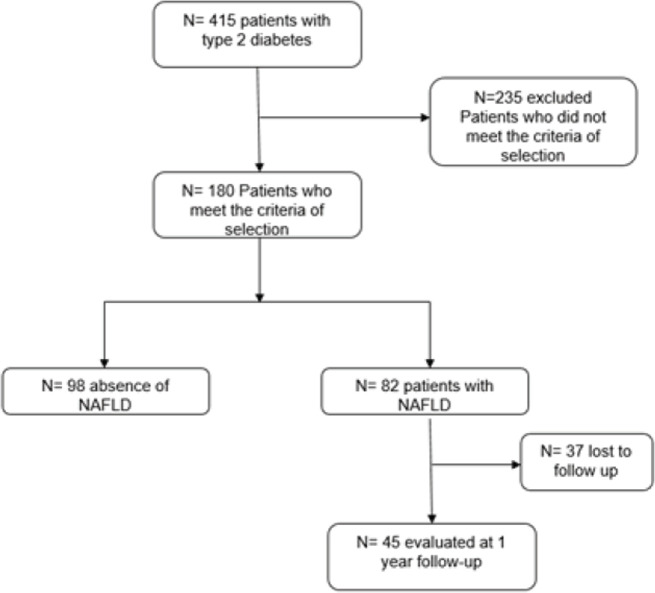
The study population.

We included all adult patients with type 2 diabetes aged between 18 and 90 years, followed-up at our University Hospital Center, who underwent NAFLD screening, and had complete medical records. Patients were excluded for known liver pathology (e.g., alcoholic, viral, drug or autoimmune hepatitis, primary biliary cirrhosis, primary sclerosing cholangitis, hemochromatosis), patients with other pathologies that could be responsible for hepatic steatosis (e.g., hypothyroidism, severe undernutrition, hypopituitarism), pregnancy and breastfeeding, active smoking, and risky alcohol consumption (> 30 g/day for men and > 20 g/day for women). From 415 patients with T2DM that were followed-up during the study period, we included 180 patients following the inclusion and exclusion criteria. The medical data of patients with NAFLD who continued their follow-up (45 patients) were collected at one-year to evaluate the clinical and biological changes after the multidisciplinary management circuit (Figure 1).

### 2.3 Study protocol

At baseline, the included patients underwent a physical examination, blood test, including a lipid profile, liver enzyme analysis, and an HbA1c assessment. The screening for NAFLD was made by performing an abdominal ultrasound. The noninvasive quantification of hepatic steatosis and fibrosis consisted of evaluating fibrosis biomarkers (NAFLD Fibrosis Score, Fibrosis-4), and performing elastography. The diagnosis of NAFLD/NASH was confirmed histologically using an ultrasound-guided liver biopsy. We also performed necessary assessments for macrovascular and microvascular complications.

### 2.4 Outcomes

The primary outcome was to determine the prevalence of NAFLD and to describe the characteristics of its association with diabetes mellitus type 2 using comparison between patients with and without NALFD. The secondary outcome was to evaluate the change from baseline to one-year follow-up on certain parameters (HbA1c, body mass index, liver tests, and hypertriglyceridemia) for patients who continued their follow-up.

### 2.5 Statistical analysis

We used the Statistical Package for the Social Sciences, version 21 (IBM, Armonk, NY) for all analyses. The relationship between NAFLD and certain factors (e.g., obesity, metabolic syndrome, hypertension, dyslipidemia) were analyzed using the student t-test and the chi-square test. A critical value of p < 0.05 indicated statistical significance.

## 3 Results

We collected data from 180 patients with a mean age of 60 ± 11 years (majority female with female-to-male ratio of 2). The mean duration of diabetes progression was 9.2 ± 7.3 years with a range of 1 to 34 years. Patients were treated in most cases with a combination of insulin and oral antidiabetic drugs (37.2%). The initial mean HbA1C was 10.4 ± 2.3%. Macrovascular complications were observed in 31.1% of cases with a predominance of coronaropathy (62.5%); we found hypertension in 47.2% of patients. We diagnosed microvascular complications in 34.4% of patients. Obesity and overweight were found in 36.1% and 28.3% of patients respectively. Metabolic syndrome was present in 70.5% of cases (Table 1).

**Table 1.** Comparison of patients with and without NAFLD

	NAFLD + (n=82)	NAFLD – (n=98)	p
Age (years)	60 ± 11	60±11,5	**0,99**
Diabetes duration (years)	9 ,5±7,6	8,8±6,9	0,52
Obesity (%)	52	13	**<0,001**
Abdominal Obesity (%)	54	13	**<0,001**
Hypertension (%)	53	32	**0,036**
Dyslipidemia (%)	47	27	**0,04**
Hypertriglyceridemia (%)	40	21	**0,028**
Metabolic Syndrome (%)	83	44	**<0,001**
HbA1C mean ± SD	10 ± 2,1	10,7± 2,5	0,15
Microvascular Complications (%)	32	30	0,39
Macrovascular Complications (%)	34	22	**0,04**

Data is expressed in n (%) or mean (SD). HbA1c = hemoglobin A1c. The comparisons were analyzed using t-student test and chi-square test. A value of p<0.05 indicated significance.

No clinical signs of liver injury were confirmed. Overall, 25 patients (30.5%) had perturbed hepatic laboratory tests – cytolysis (40%), cholestasis (44%), and hyperbilirubinemia (16%). Non-invasive hepatic biomarkers (FIB4 and NAFLD score) indicated significant fibrosis in 18.3% of cases. All patients underwent hepatic ultrasonography which showed signs of NAFLD in 45.6% of cases.

Altogether, 21% of patients underwent an elastography evaluation, revealing severe fibrosis or cirrhosis in 15.4% of patients. Liver biopsy was indicated after analysis of the clinical data, liver testing, and non-invasive evaluation of hepatic fibrosis, suspected of NASH, it could be performed in 4 patients and allowed to note a histological confirmation of NASH in 3 patients with S3A2F3 SAF score in 2 patients. The overall prevalence of NAFLD was 45.6%.

Patients with hepatic steatosis had a statistically significant incidence of obesity, metabolic syndrome, hypertension, dyslipidemia, macrovascular complications and hypertriglyceridemia, compared with patients with T2DM without NALFD (Table 2).

**Table 2.** One-year follow-up of patients with T2DM and NAFLD

	At inclusion (n=82)	Evolution after 1 year (n=45)
HbA1c mean ±SD	10 ± 2,1	7,3± 0,8
BMI mean ±SD	30,7 ±5,7	29,3± 3,5
Abnormal liver test (%)	15,3	4,2
Hypertriglyceridemia (%)	31,8	19,1

Data is expressed in n (%) or mean (SD). HbA1c = hemoglobin A1c. The comparisons were analyzed using t-student test and chi-square test. A value of p<0.05 indicated significance.

The management of NAFLD was based on a multidisciplinary approach with coordination between the Endocrinology-Diabetology-Nutrition and Hepato-Gastroenterology departments. In this report, the management of NAFLD in patients with T2DM consisted of applying dietary interventions with physical activity, evaluation and adaptation of anti-diabetic and lipid-lowering drugs, assessment of degenerative complications, research and management of co-morbidities and associated cardiovascular risk factors (obesity, dyslipidemia, hypertension, etc.). All patients with NAFLD were referred for a “specialized liver steatosis consultation” for non-invasive fibrosis assessment, investigation of NAFLD progression or aggravation, and follow-up.

A one-year follow-up evaluation was performed for 54.94% of our patients with NAFLD, who followed the multidisciplinary management circuit. The rest of the patients were lost to follow-up. We noted a substantial improvement in glycemic control, liver and lipid tests, and a slight improvement in body mass index.

## 4 Discussion

The association of T2DM and NAFLD is bidirectional and complex, involving pro-inflammatory and profibrotic phenomena, as well as insulin resistance leading to an increased risk of cardiovascular events [12]. In the presence of steatosis, instant clinical investigation for features of metabolic syndrome, insulin resistance, and T2DM is required because it is closely associated with insulin resistance. In contrast, in patients with T2DM, the evaluation of NAFLD or NASH is mandatory [2]. The increasing prevalence of this combination makes it a rising public health problem.

In this current study of 180 patients with T2DM, the overall prevalence of NAFLD on ultrasonography was 45.6%. This prevalence approaches the ones reported in American, Indian, and Algerian studies, which were 55.7%, 49% and 37.4% respectively [13-15]. Hickman et al. [16] reported an elevated prevalence of 65% in Australian patients with T2DM and obesity. The prevalence of NAFLD in patients with T2DM differs from one study to another, this disparity seems to be related to two parameters – the characteristic of the studied population and the methods used for diagnosis.

Ultrasonography has a sensitivity and specificity of 83% and 100%, respectively, for the diagnosis of NAFLD [17]. However, this means of imaging only can detect steatosis when it is above 20% without quantifying it. Nonetheless, it is recommended to use hepatic ultrasound to screen for NAFLD as a first line method considering its availability and affordability [18]. In this light, we used ultrasonography, as is done in other studies [13,16].

Other means of imaging can be used to evaluate fatty liver; however, they are costlier. Contrast-enhanced computed tomography has a sensitivity and specificity of 86% and 87% and can provide a semi-quantitative evaluation of liver steatosis [19]. MRIPDFF (magnetic resonance imaging-proton density fat fraction) and magnetic resonance spectroscopy are the only techniques of imaging that can offer precise diagnosis and quantification of fatty liver. However, they are used less often because of their high cost and limited accessibility [20].

The literature suggests that patients with NAFLD are generally asymptomatic [3]. Symptoms can occur in advanced stages of metabolic steatosis (cirrhosis and hepatocellular carcinoma) [21]. We made the same observation throughout our study.

NAFLD often causes elevated transaminases in patients with T2DM. Nonetheless, other pathologies frequently related to type 2 diabetes mellitus, such as hepatitis C virus (HCV) infection and hemochromatosis, should be eliminated before linking the abnormalities of transaminases to NAFLD [2,3].

The non-invasive quantification of liver steatosis and fibrosis assist the selection of candidates for liver biopsy. The presence and severity of fibrosis has a major prognostic value for patients with T2DM and NAFLD. Its evaluation is strongly suggested by many authors [3]. In our study, we used two biomarkers of liver fibrosis – the NAFLD Fibrosis Score and the FIB-4. These biomarkers have a strong predictive negative value in eliminating advanced fibrosis (stages F3-F4) [22,23]. Transient elastography of FibroScan is another non-invasive method for the assessment of hepatic fibrosis. In our study, the use of FibroScan revealed an advanced fibrosis in 15.78% of cases. This tool has a strong predictive negative value; moreover, it is available, reproducible, and easy to use [24].

Liver biopsy is the standard of diagnosis and quantification of steatosis, fibrosis and necro-inflammatory activity in patients with T2DM and NAFLD [25]. It helps to determine the presence of NAFL or NASH. Based on the European recommendations of clinical practice for the study of liver, diabetes and obesity, we decided to perform liver biopsy in 10 patients after thorough assessment of their medical data, liver biomarkers, and transient elastography. However, considering the costs and the risks of this method, liver biopsy was only performed in four patients in our study. Hazlehurst et al. [26] reported that NAFLD can be found in up to 70% of patients with T2DM, but the prevalence of histologically confirmed NASH remains less than 20%.

Regarding risk factors of NAFLD in T2DM, patients with NAFLD in our study were not statistically older and did not have a longer diabetes history compared to patients without NAFLD (Table 2). However, the presence of metabolic syndrome in our patients with NAFLD had a statistical significance. In fact, many authors consider NAFLD as a spectrum of visceral obesity and metabolic syndrome [27]. Obesity and dyslipidemia have been proven to be factors of initiation and progression of NAFLD in patients with T2DM [1,28]. We made the same observation in our study (Table 2).

Our patients with NAFLD had a statistically significant risk of hypertension and macrovascular complications. According to the literature, NAFLD increases the risk of cardiovascular events in patients with T2DM independently of the presence of other cardiovascular risk factors [29]. Moreover, the abnormal hepatic accumulation of lipids enhances insulin resistance and causes weight gain [30].

T2DM constitutes a factor of progression of NAFLD to severe forms of NASH with advanced fibrosis and higher risk of cirrhosis or hepatocellular carcinoma [31]. In addition, some authors suggest that the mortality of patients with T2DM and NAFLD is related to cancers and cardiovascular events, because NAFLD is associated with an increased risk of heart failure, atrial fibrillation, and coronaropathy [10,29].

Currently, no medical treatment has been authorized for the management of NAFLD in patients with T2DM. However, some studies report the benefits of some anti-diabetic drug classes on hepatic laboratory tests, metabolic syndrome, and histologic injuries of NAFLD/ NASH [18,32,33].

Dietary interventions and physical activity have been recommended to all our patients. Some studies demonstrate that caloric restriction and physical activity improve the metabolic features and histological injuries of NAFLD/NASH [2,31]. The use of certain treatments that have a direct impact on NAFLD such as ursodeoxycholic acid in patients with T2DM is limited. The other treatment options used regularly, are in fact, anti-diabetic drugs, that improve insulin resistance and glycemic control, and thus, impact NAFLD [2,26].

The use of metformin in patients with T2DM and NAFLD can modestly improve cytolysis, without a direct impact on steatosis and the progression of liver fibrosis [34,35]. Data related to the impact of dipeptidyl peptidase-IV (DPP-VI) inhibitors is still unclear. Yet, Svegliati-Baroni G et al. [36] noted in their prospective study that DPP-VI inhibitors reduced the rate of triglycerides in the liver, an effect that was measured with MR spectroscopy.

The pilot LEAN (Liraglutide safety and efficacy in patients with non-alcoholic steatohepatitis) study is a randomized double-blind, multicentric study, that used Liraglutide versus placebo during 48 weeks on 52 patients with obesity and insulin resistance, including one-third of patients with T2DM [32]. In this study, a significant resolution of histologic injuries was observed in the group treated with Liraglutide, without worsening the progression of fibrosis. An improvement on biological assessment and insulin resistance has been noted as well.

Sodium-glucose co-transporter-2 (SGLT2) inhibitors were not used with our patients, but some studies suggest that SGLT2 inhibitors have a positive impact on glycemic control, weight loss, triglycerides and cytolysis [37].

The limitations of our study include the small number of patients who continued their follow-up after one year. Another issue is the small number of patients who underwent liver biopsy to confirm histologically the diagnosis of NASH, although it remains invasive.

The strength of our study is the fact that our study is the first study that provides an estimation of NAFLD in patients with type 2 diabetes in Morocco, and more precisely, in the eastern region. Our study gives an insight into the risk factors associated to NAFLD in our population compared to other studies.

### 4.1 Conclusion

The combination of NAFLD and type 2 diabetes mellitus is common. It is often found in patients with obesity or metabolic syndrome. The presence of NAFLD can be responsible for significant morbidity and important cardiovascular risks in patients with diabetes mellitus type 2 [10]. Current treatment options for NASH are limited. A few studies suggest new agents such as Glucagon-like Peptide-1 (GLP-1) agonists as promising drugs, which have demonstrated significant improvement in histological resolution of NASH [32].

## Abbreviations

NAFLD: Non-alcoholic fatty liver disease

T2DM: Type 2 diabetes mellitus

NASH: Non-alcoholic steatohepatitis

NAFL: Nonalcoholic fatty liver

HCV: Hepatitis C virus

FIB-4: Fibrosis-4 index
